# Diffusion Spectrum Imaging Shows the Structural Basis of Functional Cerebellar Circuits in the Human Cerebellum *In Vivo*

**DOI:** 10.1371/journal.pone.0005101

**Published:** 2009-04-02

**Authors:** Cristina Granziera, Jeremy Dan Schmahmann, Nouchine Hadjikhani, Heiko Meyer, Reto Meuli, Van Wedeen, Gunnar Krueger

**Affiliations:** 1 Department of Neurology, Centre Hospitalier Universitaire Vaudois, Lausanne, Switzerland; 2 Department of Neurology, Massachusetts General Hospital, Harvard Medical School, Boston, Massachusetts, United States of America; 3 Athinoula A. Martinos Center for Biomedical Imaging, Massachusetts General Hospital, Harvard Medical School, Charlestown, Massachusetts, United States of America; 4 Brain Mind Institute, Ecole polytechnique fédérale, Lausanne, Switzerland; 5 Siemens AG, H IM MR PLM AW Neurology, Erlangen, Germany; 6 Department of Radiology, Centre Hospitalier Universitaire Vaudois, Lausanne, Switzerland; 7 Siemens Schweiz AG, Healthcare Sector IM&WS S, Renens, Switzerland; Julius-Maximilians-Universität Würzburg, Germany

## Abstract

**Background:**

The cerebellum is a complex structure that can be affected by several congenital and acquired diseases leading to alteration of its function and neuronal circuits. Identifying the structural bases of cerebellar neuronal networks in humans *in vivo* may provide biomarkers for diagnosis and management of cerebellar diseases.

**Objectives:**

To define the anatomy of intrinsic and extrinsic cerebellar circuits using high-angular resolution diffusion spectrum imaging (DSI).

**Methods:**

We acquired high-resolution structural MRI and DSI of the cerebellum in four healthy female subjects at 3T. DSI tractography based on a streamline algorithm was performed to identify the circuits connecting the cerebellar cortex with the deep cerebellar nuclei, selected brainstem nuclei, and the thalamus.

**Results:**

Using *in-vivo* DSI in humans we were able to demonstrate the structure of the following cerebellar neuronal circuits: (1) connections of the inferior olivary nucleus with the cerebellar cortex, and with the deep cerebellar nuclei (2) connections between the cerebellar cortex and the deep cerebellar nuclei, (3) connections of the deep cerebellar nuclei conveyed in the superior (SCP), middle (MCP) and inferior (ICP) cerebellar peduncles, (4) complex intersections of fibers in the SCP, MCP and ICP, and (5) connections between the deep cerebellar nuclei and the red nucleus and the thalamus.

**Conclusion:**

For the first time, we show that DSI tractography in humans *in vivo* is capable of revealing the structural bases of complex cerebellar networks. DSI thus appears to be a promising imaging method for characterizing anatomical disruptions that occur in cerebellar diseases, and for monitoring response to therapeutic interventions.

## Introduction

The cerebellum is a complex structure that plays a major role in motor control [1] as well as in cognitive-emotional processing [2], [3]. Knowledge regarding structure of the human cerebellum is essential for understanding the functional consequences of congenital and acquired neurological diseases of the cerebellum including sporadic and hereditary ataxias, the consequences of focal lesions such as stroke, and the cerebellar component of neuropsychiatric diseases including schizophrenia, Asperger's syndrome and autism [4]–[8].

Investigations of the gross anatomy of the human cerebellum date back to the 18^th^ century [9]–[11] and have been further elaborated upon in recent human MRI atlases [12]–[15]. In contrast, knowledge of intrinsic neural circuits of the cerebellum and extracerebellar connections with spinal cord, brainstem and cerebral hemispheres has been derived exclusively from tract tracing studies and physiological investigations in animals because there has been no method available for the study of these pathways and circuits in the human brain [16]–[22]. Recent developments in MRI technology, however, have enabled the study of the anatomical basis of cerebellar circuits in humans using diffusion tensor imaging (DTI) methodology. Some advances have been made using DTI [23] but the underlying diffusion tensor model has intrinsic limitations that permit only partial visualization of cerebellar white matter tracts, and limited capability to reveal complex anatomical details of the cerebellar circuits [23].

In contrast, diffusion spectrum imaging (DSI), a high angular resolution diffusion technique [24], is able to define more complex structures such as crossing fibers. DSI has proven useful in studying the fiber tracts and connections of the human cerebrum and cerebellar systems *in vitro* [25], [26]. We hypothesized that DSI would yield new insights into the organization of the human cerebellum *in vivo*. Specifically, we tested the hypothesis that the connections of the human cerebellum *in vivo* would reflect those identified in the experimental animal, and be consistent with findings of the limited published post mortem studies to date.

## Methods

### Image acquisition and DSI tractography reconstruction

Four healthy female participants (age: 26±4 yrs) underwent magnetic resonance DSI in a commercial 3T scanner (Trio a Tim System, Siemens, Erlangen, Germany) using a 32-channel head helmet coil. The study was approved by the Institutional Review Board of Siemens AG, Healthcare Sector, Imaging, Magnetic Resonance, Process Lifecycle Management (H IM MR PLM, Erlangen, Germany). All subjects provided written informed consent prior to the imaging session. DSI was performed using a single-shot spin-echo echo-planar imaging (EPI) product sequence and the following parameters: TR/TE = 6600/138, FoV = 212 mm, 34 slices, 2.2 mm isotropic resolution, GRAPPA = 2, 258 diffusion directions covering a half q-space 3D grid with radial grid size of 5, b(max) = 8000 s/mm^2^ and one image acquired at b = 0 s/mm^2^ (referred to here as *b0-image*), total acquisition time = 28:44 min. DSI scans centered in the cerebellum were acquired twice and averaged subsequently. Diffusion encoding was performed using a bipolar encoding scheme to minimize distortion effects due to residual eddy-current effects introduced by the diffusion gradient pulses [27]. High and low b-value scans were interleaved to qualitatively assess subject motion. For anatomical reference a whole brain high-resolution MPRAGE was acquired using the parameters described in the ADNI protocol (http://www.loni.ucla.edu/ADNI/Research/Cores/ADNI_Siemens_3T_TrioTimVB13.pdf) (TR: 2400 ms, TE: 3.59 ms, 0.8 mm isotropic resolution, FOV256×256). DSI reconstruction was performed with Diffusion toolkit [28] using data from single DSI acquisitions and from the averaged raw images. Subsequently, DSI tractography was performed based on a FACT-like streamline algorithm [29] using the TrackVis software [28]. We seeded a path for every orientation density function (odf) max vector at every voxel, extending the path along the vector of least curvature in a new voxel, and stopping the process if this curvature ≥35°. The colour-coding of the obtained fibers is based on standard RGB code applied to the vector at every segment of each fiber. Blue indicates the rostro-caudal direction; red the medio-lateral plane; and green the dorso-ventral orientation.

### Region of interest (ROIs) selection

We used the TrackVis 3-D tool to select the ROI in b0 images. The anatomical structure corresponding to the desired ROIs was initially localized in MRI atlases of the cerebellum [12], [13], subsequently identified in the MPRAGE images, and then selected in the co-registered b0 dataset. The following regions were defined as seed-point for the analysis: 1) the inferior olivary nucleus; 2) deep cerebellar nuclei; 3) ventrolateral region of thalamus; 4) red nucleus; and 5) the superior cerebellar peduncle (SCP), 6) middle cerebellar peduncle (MCP), and 7) inferior cerebellar peduncle (ICP). The pathways identified by performing tractography through the ROIs were compared with known anatomical pathways as defined in human gross anatomy texts [30] and in connectional studies in experimental animals [31]–[33].

## Results

**Figure 1:**
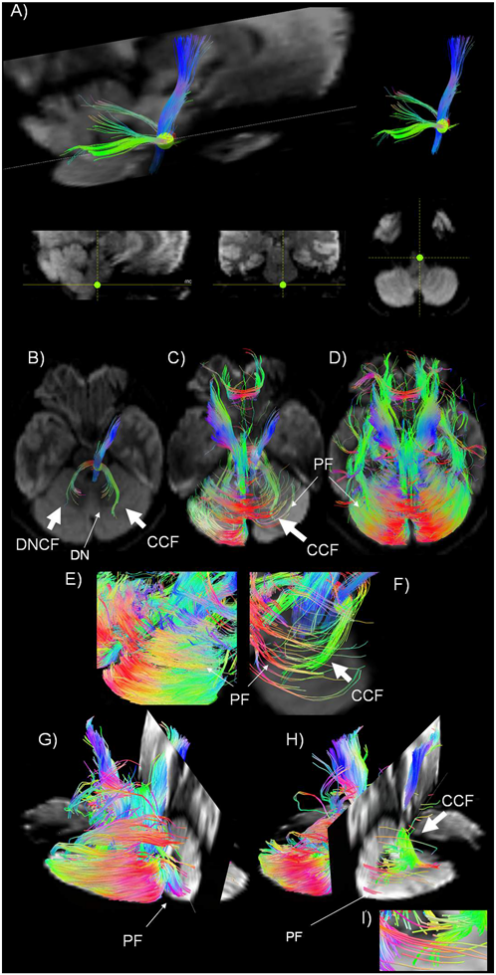
Olivary-cerebellar cortex connections and olivary-dentate-olivary loop. A) Sagittal tractography view, in a DWI image background on the left, showing the connections between (i) the Inferior olivary nucleus (green region of interest -ROI) and the cerebellar cortex (light bright green fiber trajectories) and (ii) the Inferior olivary nucleus and the Dentate nucleus (dark green fiber trajectories). The ROI in the Inferior olive is shown in the sagittal, coronal, and axial planes in the images at bottom (from left to right). B), C) and D) Axial tractography view, in a DWI background, showing: B) the connections between (i) the Inferior olivary nucleus (green ROI) and the dentate nucleus (DN) through Dentate nucleus climbing fibers (DNCF) and (ii) the connections between the inferior olivary nucleus (green ROI) and the cortex through cortical climbing fibers (CCF); C) the intersection between the CCF and parallel fibers (PF) from the granule cell axons. D) PF in the cerebellar cortex. E) and F) Tractography magnification of an axial view of the cerebellar cortex, in DWI background. PF and on the left, PF intersecting CCF. G), H), I) 3D tractography view, in a DWI background, showing: G) some PF traversing the cerebellar cortex. H) PF crossing CF that are oriented perpendicular to them. I) Higher magnification of H).

The following pathways and connections were consistently observed and visualized in all four subjects:

1. *Connections between the inferior olivary nucleus and the cerebellar cortex, and collaterals to the deep cerebellar nuclei (* *figure 1 A* *and B-I)*.

**Figure 2:**
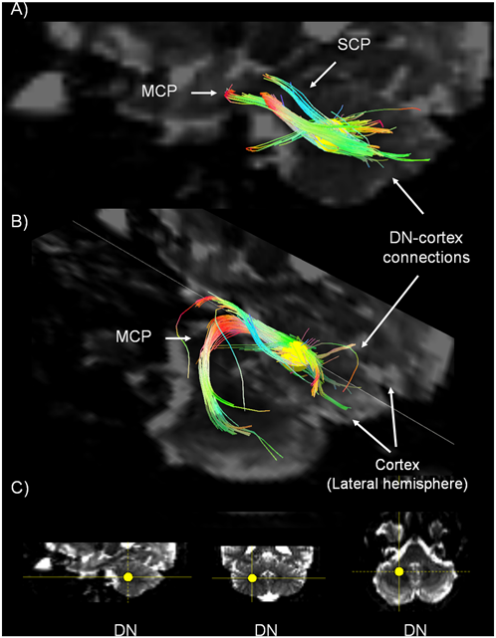
Dentate nucleus connections. A) Sagittal view and B) sagittal and axial 3D view of the Dentate nucleus (DN - see yellow ROI) in a b0 background image. We show: (i) a subset of connections between DN and the cerebellar cortex (lateral hemisphere) and (ii) fibers identified by the ROI in the DN travelling in the MCP and the SCP. C) 3D localization of the Dentate Nucleus (yellow ROI) in a b0 background image in sagittal, coronal, and axial views (from left to right).

We positioned a ROI in an area corresponding to the inferior olivary nucleus, situated in the rostral medulla oblongata between the pyramidal tract and the lateral reticular nucleus (green ROI, figure 1 A). Tracking from this ROI, we identified a pathway entering the cerebellum through the ICP and connecting to: 1) the dentate nucleus (light green, figure 1 A) and 2) the cerebellar cortex (dark green, figure 1 A). This pathway is consistent with the trajectory of the olivocerebellar climbing fiber system.

The collaterals to the dentate nucleus constitute a reciprocal loop connecting the inferior olivary nucleus with the deep cerebellar nuclei (olivary-dentate-olivary loop, figure 1 B). Fiber solutions that do not connect to the dentate nucleus reach the cerebellar cortex (olivary-cerebellar cortex connections, figure 1 B- I), crossing at right angles with fibers in the cortex that have a location and orientation consistent with parallel fibers. This intersection of fiber trajectories in the cerebellar cortex could be identified using the 3D display of the TrackVis software [28].

From the same ROI, we also visualized fiber tracts within the pyramidal tract (blue, figure 1 A), because of the close proximity of the inferior olivary nucleus and the pyramid in the medulla.

**Figure 3:**
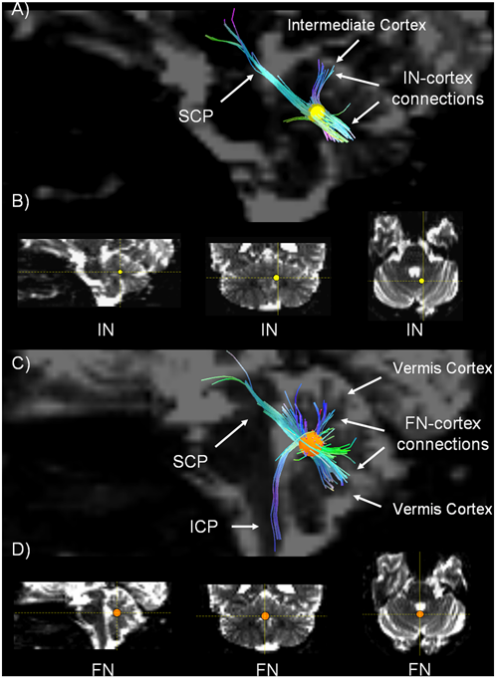
Interpositus and Fastigial nuclei connections. A) Sagittal view of the region containing the Globose and Emboliform nuclei (interpositus nucleus (IN) yellow ROI), in a b0 background image. We show (i) the connections between the IN and the cerebellar cortex (intermediate cortex) and (ii) the fibers that link these deep cerebellar nuclei with the SCP. B) 3D localization of the location of the Globose and Emboliform nuclei (yellow ROI) in a b0 background image in the sagittal, coronal, and axial planes, from left to right. C) Sagittal view of the Fastigial nucleus (FN) (see orange ROI), in a b0 background image. We show (i) the connections between the FN and the cerebellar cortex (vermis) and (ii) the fibers that link the FN with the SCP and ICP D) 3D localization of the FN (see orange ROI) in a b0 background in bottom figures in the sagittal, coronal, and axial planes, from left to right.

2. *Connections between the cerebellar cortex and the dentate nucleus (* *figure 2 A, B* *), emboliform and globose nuclei (* *figure 3 A, B* *), and the fastigial nucleus (* *figure 3 C, D* *)*.

The Purkinje cells in the cerebellar cortex project to those parts of the deep cerebellar nuclei that are closest to them, conforming to a zonal, parasagittal orientation [16], [17], [19], [34], [35]. Thus, the lateral hemispheres project to the dentate nucleus (figure 2 A, B); the intermediate cortex project to the globose and emboliform nuclei (figure 3 A); and the vermis projects to the fastigial nucleus (figure 3 C).

We were able to show only a subset of the fibers expected to connect the dentate nucleus with the lateral hemispheres of the cerebellar cortex, most likely a consequence of signal-to-noise ratio (SNR) and angular resolution limitations. Similarly, we were unable to visualize the predicted connections between the fastigial nucleus and lobules IX-X of the vermis [33].

3. *Efferent projections from the deep cerebellar nuclei conveyed in the three cerebellar peduncles (* *figures 2 A, B, C* *and* *3 A–D* *)*.

After identifying a ROI in the area corresponding to each deep nucleus (figure 2 and 3), we visualized fiber trajectories travelling in 1) the SCP and the MCP linked to the dentate nucleus (figure 2 A, B); 2) the SCP from the interpositus nucleus (figure 3 A, B); and 3) the SCP and ICP from the fastigial nucleus (figure 3 C, D). In order to better visualize the characteristic morphology of the MCP, we also obtained a 3D view showing this tract coursing around the basis pontis in the axial plane (figure 2 B).

**Figure 4:**
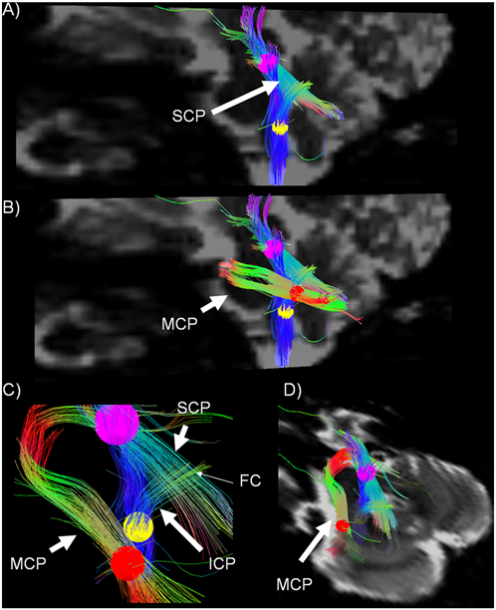
The three cerebellar peduncles. Sagittal b0 image showing the superior (SCP- see purple ROI) and Inferior cerebellar peduncles (ICP – see yellow ROI) crossing in the cerebellar white matter core. From the yellow ROI in the brainstem, a pathway connecting to the diencephalon is also tracked B) Sagittal b0 image showing the middle cerebellar peduncle (MCP – see red ROI & white arrow). C) Sagittal view, no background showing the fiber-crossing region (FC) between the inferior and the superior cerebellar peduncles (white arrows) at higher magnification. D) Coronal b0 image showing the precise location of the ROI used to seed the MCP (red ROI, white arrow).

**Figure 5:**
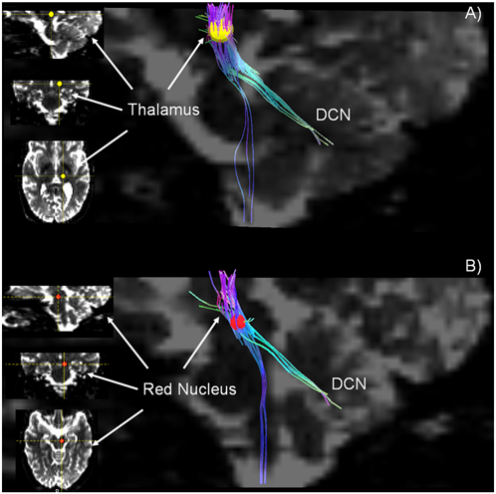
Thalamic – and Rubro-cerebellar connections. A) Sagittal b0 image showing cerebellar connections with thalamus (green). The ROI (yellow) is positioned in the ventro-lateral thalamus, as seen on the left in sagittal, coronal and axial slices, from above to below. Some fiber trajectories connecting the thalamus to the brainstem are also shown (blue fiber tracts). B) The 3D localization of the ROI (red) in the red nucleus is shown at left in the sagittal, coronal and axial slices, from above to below. The sagittal b0 image shows the rubro-cerebellar connections (green). Some fiber trajectories connecting the red nucleus to the brainstem are also identified (blue fiber tracts).

4. *Complex crossing intersections between the SCP and the ICP (* *figure 4 A–C* *), and the intersection between the MCP and the ICP (* *figure 4 B, C, D* *).* We positioned 3 ROIs along the 1) SCP (upper pons, figure 4 A–D) 2) MCP (lower pons, figure 4 B–D) and 3) ICP (medulla oblongata, figure 4 A–C). In this way, we visualized the intersection between the SCP and the ICP (figure A–C) and the 3D spatial relationship between the MCP and the SCP/ICP respectively (figure 4 B, D).

5. *Connections that link the deep cerebellar nuclei with the red nucleus and thalamus (ventro-lateral region) (* *figure 5 A and B* *).*

When the ROI is placed in the region of the ventro-lateral thalamus, fiber trajectories decussate to the contralateral cerebellum and penetrate the medullary core of the cerebellum in the region of the deep cerebellar nuclei. Some fiber trajectories that emanate from the thalamic ROI towards cerebellum cannot be traced to the cerebellum itself. Other fiber solutions identified by the thalamic ROI continue into the spinal cord, likely reflecting ascending spino-thalamic fiber systems.

The ROI placed in the red nucleus (figure 5 B) results in a contingent of fibers that courses into the cerebellum, consistent with the known efferent pathway leading from the interpositus nucleus via red nucleus to thalamus. Other fiber solutions course towards spinal cord. This may reflect fibers in the rubrospinal tract, but it is also possible that it reflects corticospinal fibers in the cerebral peduncles adjacent to the ROI in the red nucleus.

## Discussion

In this study, we used Diffusion Spectrum MRI to test whether it is feasible to examine the intrinsic and extrinsic cerebellar circuits in the living human brain. Such ability represents a necessary step in defining pathological anatomy of the spinocerebellar and other ataxic disorders, in describing the cerebellar component of neuropsychiatric illness, and developing biomarkers for disease progression and modifying interventions. We show, for the first time in humans, that DSI has the capacity to elucidate the structural basis of neural circuits in the human cerebellum *in vivo*.

We improved the intrinsic low-sensitivity of the DSI method by optimizing the acquisition protocol and by using a 32-channel head coil array at 3T to a level that allowed us to reconstruct and visualize cerebellar circuits and pathways.

The study of cerebellar connectional networks is only partially possible with DTI techniques, because DTI suffers from the limitation of being unable to resolve the convergence of multiple fiber bundles/connections into relatively small structures, as occurs in the white matter in the medullary core of the cerebellum, and within the cerebellar cortex itself [23], [36]. DSI, a high angular resolution method which images complex distributions of intra-voxel fiber-orientations, overcomes this limitation of the DTI technique and has the demonstrated capability of identifying fiber crossings within neural structures [24]. This is exemplified by the DSI demonstration of long association fibers pathways in the monkey cerebral hemisphere, observations that were supported by comparison with the results of tract tracing studies using the autoradiographic technique [25]. Further, DSI is sufficiently powerful to map regions of fiber-crossing not only in cerebral white matter, but also in the basis pontis and the cerebral and cerebellar cortices in monkey and human brains post-mortem [26]. It is noteworthy that related techniques such as q-ball imaging may lead to very similar results [37], [38]. However, since DSI represents the most general approach to disentangle complex structures it was used in this investigation. To our knowledge, no attempt has been made to map cerebellar connectivity networks with DSI in humans *in vivo*.

Combining the ROI-based DSI tractography with high-resolution anatomical images, we were able to visualize the olivo-cerebellar circuits in humans *in vivo*. The fiber tracks that we demonstrated linking the inferior olivary nucleus with the cerebellar cortex and with collaterals to the deep cerebellar nuclei (olivary-cerebellar nucleus-olivary loop), likely correspond to the course and connectional patterns of the climbing fibers that originate in the inferior olivary nucleus. We were also able to identify essential elements of the intrinsic cerebellar circuitry: the cortico-nuclear projection between deep cerebellar nuclei (fastigial, interpositus and dentate) and the cerebellar cortex; and the intrinsic cerebellar cortical circuitry, characterised by fiber solutions corresponding to the granule cell axons' parallel fibers that traverse the long axis of the cerebellar folium, intersecting with the perpendicularly arranged fiber-tracks consistent with climbing fibers of the olivocerebellar system traced from the ROI in the inferior olive [39]. From tract tracing studies and physiological investigations in animals [16]–[18], [34], it is known that climbing fibers (CF) originate from neurons in the inferior olivary nucleus and terminate around the proximal dendrites of the Purkinje cell (PC). Parallel fibers (PF), axons of the granule cells, synapse with the distal dendrites of the PC. The CF and PF thus constitute part of the connectivity substrate of the molecular layer in the cerebellar cortex. DSI cannot reach to the level of the synapse, but it does identify the perpendicular orientation of these two cerebellar afferent fiber systems that intersect in the cerebellar molecular layer, and it does so *in vivo*.

We show the extracerebellar pathways that are linked with the deep cerebellar nuclei in the SCP, MCP and ICP. The capability of the DSI method to resolve crossing fibers also makes it possible to provide a visualization in human *in vivo* of the complex fiber intersections between the three cerebellar peduncles, previously shown in tracing studies [16], [17], [19]. Such a demonstration has not been possible using DTI.

We have also provided evidence of the ability using DSI to identify the thalamo-cortical pathway in humans by seeding the ventrolateral region of thalamus, and the rubro-cerebellar connection by placing the ROI seed in the red nucleus.

### Advantages and limitations of the methodology

Optimizations of the acquisition protocol and the use of a dedicated 32-channel coil with excellent SNR properties based on Wiggins et al. [40], aimed at compensating for some of the unfavorable SNR properties in high b-value diffusion MRI, including averaging of consecutive scans [41]. Using this state-of-the-art methodology and TrackVis 3D for interactive visualization of fiber trajectories, we could map complex cerebellar tracts and connectional pathways.

We note that accuracy of the tractography method is user dependent because reconstruction threshold, turn angle, and mask threshold have to be adapted according to scan parameters and image properties. To address this limitation, we applied identical tracking parameters to all the data processing.

Further, despite the advanced technology and methods used for DSI acquisition, in some cases we could only partially map the cerebellar circuit of interest. For example, we could visualize only a subset of the fibers tracks connecting the dentate nucleus ROI to the cerebellar cortex.

We hypothesize that this could have happened for three reasons: first, the dentate nucleus is located deep within the cerebellum. Due to the coil design the highest sensitivity and SNR is provided in the superficial layers [40], whereas regions closer to the center of the brain, such as the deep cerebellar structure are imaged with a lower SNR rendering our method less sensitive in these regions. Second, the dentate nucleus is a small structure where fiber trajectories potentially diverge at smaller angles than the angular resolution power of our DSI acquisition scheme.

For similar reasons, we could not track the complete path connecting the ROI in thalamus and brainstem to the cerebellar cortex via the deep cerebellar nuclei. Two separate fiber bundles belonging to these pathways had to be delineated: first from the ROI in thalamus and brainstem to the cerebellar deep nuclei, and then from the deep nuclei to the cortex. Third, subject motion as well as brain pulsation originating from cardiac and respiration cycles may limit DSI tracking. Future iterations of this DSI approach could be enhanced by combining specific higher SNR and angular resolution at reduced scan times, as well as adding image registration methods.

It is notable that all subjects tolerated the scan protocol with minimal or no evidence of motion according to qualitative visual inspections of the low b-value images, i.e. motion is evaluated to be in the sub-voxel regime and thus of minimal influence for our analysis.

In sum, we were able for the first time to visualize human cerebellar circuits *in vivo* non-invasively using DSI. We demonstrate pathways and connections that are in general agreement with histological tract tracing studies in animal models. *In vivo* DSI of the cerebellum has the potential to introduce new insights into the pathophysiology of neurological and neuropsychiatric diseases, and to provide anatomical and connectional biomarkers of cerebellar disease.
